# GPs' experiences of a collaborative care model for patients with common mental disorders who need sick leave certification: a qualitative study

**DOI:** 10.3399/BJGPO.2022.0042

**Published:** 2022-10-05

**Authors:** Ausra Saxvik, Irene Svenningsson, Karin Törnbom, Eva-Lisa Petersson, Cecilia Björkelund, Goda Gabartaite, Dominique Hange

**Affiliations:** 1 Primary Health Care/Department of Public Health and Community Medicine, Institute of Medicine, Sahlgrenska Academy, University of Gothenburg, Gothenburg, Sweden; 2 Research, Education, Development & Innovation, Primary Health Care, Region Västra Götaland, Sweden; 3 Department of Social Work, University of Gothenburg, Gothenburg, Sweden

**Keywords:** primary health care, general practitioners, common mental disorders, collaborative care, care manager

## Abstract

**Background:**

GPs are an important part of collaboration around patients with common mental disorders (CMD) in primary care. The Co-Work-Care model was implemented to further improve collaboration, and emphasised working more closely with patients through active dialogues among care managers, rehabilitation coordinators, and GPs. This enhanced collaborative model also included a person-centred dialogue meeting with patients’ employers.

**Aim:**

The aim of this study was to explore GPs’ experiences of the Co-Work-Care model, an organisation of collaborative care at the primary care centre (PCC) that includes a person-centred dialogue meeting in the care of patients with CMD who need sick leave certification.

**Design & setting:**

Qualitative individual and group interviews were conducted with Swedish GPs with experience of the Co-Work-Care trial where the PCC was an intervention PCC with the enhanced collaboration model.

**Method:**

GPs were sampled purposefully from different Co-Work-Care intervention PCCs in Sweden. Focus group and individual, in-depth semi-structured interviews were conducted. All interviews were analysed by systematic text condensation (STC), according to Malterud.

**Results:**

The following three codes describing the GPs’ experiences of working in the Co-Work-Care model were identified: (1) a structured work approach; (2) competency of the care manager and the rehabilitation coordinator; and (3) gaining control through close collaboration.

**Conclusion:**

Overall, GPs’ experience was that the enhanced collaboration reduced their workload and enabled them to focus on medical care. Patient care was perceived as safer and more effective. These advantages may result in higher quality in medical and rehabilitation decisions, as well as a more sustainable and less stressful work situation for GPs.

## Introduction

The return-to-work (RTW) process is often complex and dependent on different aspects, such as provided care and rehabilitation, the workplace environment, the employer, and not least the individual’s health and personal resources.^1^ How professional parties interact and how well they collaborate are also primary contributors to the RTW process. To attain a successful RTW, multidisciplinary collaboration both within health care, and between health care and the patient’s workplace, needs to be in focus for interventions.^1,2^

The rehabilitation coordinator’s function in Swedish primary care is to initiate contact with the employer and other external stakeholders, and to play a supportive role for the patient and the GP responsible for the sick leave certification during the RTW process.^3,4^ The main role of the GP is to assess and understand the patient's medical situation, in order to decide about rehabilitation, sick leave, or other interventions.^4^

CMD, including depression, anxiety, and stress-related disorders, affect 10–18% of the working population worldwide and are an increasing problem in high-income countries with well developed healthcare systems..^5^ Depression and anxiety disorders have an extensive impact on a person’s individual quality of life in terms of health, working life, and psychosocial functioning.^6^ In Sweden, CMD is one of the leading causes of sickness absence (40% of all cases) among both men and women of working age.^7^ A majority (70%) of patients suffering from CMD are diagnosed and treated in a primary care setting.^8^

The Co-Work-Care model used in the current study was based on early collaboration between a care manager, a rehabilitation coordinator, and the GP responsible for the patient’s sick leave certification.^4^ The care manager coordinates care by maintaining a supportive and regular contact with patients and aligns efforts according to patients’ individual needs.^5^ The rehabilitation coordinator has a supportive function in collaboration at the PCC and supports patients during their sick leave and RTW process.^9^ Research concerning GPs’ experiences of being a part of a rehabilitation collaboration is scarce.^10,11^ According to one study conducted in Sweden, GPs experienced relief when working with a care manager in the treatment of patients on sick leave.^12^ GPs also considered that working together with a rehabilitation coordinator led to more precise assessments of individual rehabilitation needs.^4^

The aim of this study was to explore GPs’ experiences of the Co-Work-Care model, an organisation of collaborative care at the PCC that includes a person-centred dialogue meeting in the care of patients with CMD.

## Method

### Study design

This study adheres to the Standards for Reporting Qualitative Research (SRQR) guidelines.^13^ The study consisted of one focus group discussion and two individual interviews with GPs at three different PCCs located in the south-west of Sweden. Focus group discussions allow participants to exchange different experiences and thoughts, producing rich qualitative material through these collective reflections.^14^ Individual interviews complemented these by providing more personal, in-depth information, and contents of a more private character.^15^

### The Co-Work-Care model

Experiences of working within the Co-Work-Care model were studied from the GP’s perspective. The Co-Work-Care model included an in-depth collaboration among the care manager, rehabilitation coordinator, and the GP, followed by a person-centred dialogue meeting between the patient and their employer, with the rehabilitation coordinator attending as a dialogue moderator.^4^

Initially, the patient with CMD meets the GP, who assesses the need for a sick leave certificate and refers the patient to a care manager and a rehabilitation coordinator. The in-depth collaboration between the care manager, rehabilitation coordinator, and GP is in part determined by the unique preconditions at each PCC. During the care process and collaboration between the care manager, rehabilitation coordinator, and GP, the patient’s situation becomes clearer and forms the basis for further decisions about sick leave and for the person-centred dialogue meeting.

Commonly, the care manager is involved in the patient’s situation from day one, and the rehabilitation coordinator is involved in all work-related rehabilitation collaboration surrounding the patient before and during the person-centred dialogue meeting. Depending on the size of the PCC and other practical circumstances, in some cases the same nurse has the role of being both the care manager and the rehabilitation coordinator. Further, the care manager improves accessibility and continuity for the patient. The role of the care manager is based on the patient’s specific needs over time, including support regarding medical issues and self-management. The format allows for complexity, person-centred care, and interaction.^16^

The rehabilitation coordinator provides support at the PCCs for effective collaboration in the sick leave certification and rehabilitation process. Further, the rehabilitation coordinator should coordinate workplace-related rehabilitation, as well as be a knowledge broker and adviser regarding health insurance and certificate issues and the rehabilitation plan.^4^

### The person-centred dialogue meeting

When forming the structure and content of the person-centred dialogue meeting, a theoretical framework for a traditional convergence dialogue meeting was used.^17^ The main aim of this meeting was to facilitate the patient's RTW process^18^ and thus shorten their sick leave period.^17^ However, the convergence dialogue meeting was modified to meet the preconditions at the PCCs and is thus referred to as a person-centred dialogue meeting. The person-centred dialogue meeting is based on patients’ experiences of their situations, which clarifies their needs and requirements. The main aim of the meeting is to give the patients an opportunity to sit down with their employer and the rehabilitation coordinator in a calm and neutral environment (preferably at the PCC) and to describe their situation in their own words in front of the employer. The meeting is based on the patient’s experience of their situation and includes no negotiation.^4^ The Co-Work-Care model is more extensively described in an earlier publication.^4^

### Data collection and study participants

Data were collected through one focus group discussion with seven GPs and two individual interviews; the participating GPs were from three different PCCs, which had most extensive experience of the Co-Work-Care model during January–May 2021. The focus group discussion and the individual interviews were performed jointly by two researchers (IS, ELP) from the Co-Work-Care project research group.

Participants (*n* = 10, three men and seven women) were GPs with 12–30 years of work experience, who had participated in the Co-Work-Care trial at ‘intervention’ PCCs with the enhanced collaboration model.

All interviews were conducted and audio-recorded through the videoconference program Skype. Initial reflections were written down during the sessions. The material was then transcribed verbatim and kept confidential in the researchers’ office. The researchers used an interview guide (see Supplementary file 1) during the focus group discussion and the individual interview, based on the main research question: 'When working within the Co-Work-Care model, how do you generally perceive collaboration among the GP, rehabilitation coordinator, and care manager with patients on sick leave due to CMD?' Supplementary questions were posed to further probe participants’ answers, thereby yielding an interview material that was rich in content.

### Analysis

Data analysis was performed using STC according to Malterud, which is a method for systematic thematic cross-case analysis.^19^ The analysis was a collaboration within the entire research team. KT is a social worker with a doctoral degree and has experience in psychotherapy in outpatient care. AS is a GP. ELP is an occupational therapist, and IS is a district nurse; both are associate professors with long experience in primary health care. CB is a GP and professor, and DH is a GP and associate professor. In order to grasp the essence of the data, the researcher’s own theoretical perspective and preconceptions were set aside as much as possible, as is emphasised in STC.^19^

Initially, the transcripts were read several times to get an overview of the material and to gain a preliminary view of potentially interesting codes. In the second step, meaning units that contained information about the research questions were extracted; that is, GPs’ experiences of collaboration with care managers, rehabilitation coordinators, and the person-centred dialogue with the employer.

During the third step of analysis, codes and subcodes were identified. Further, the meaning units of each subgroup were reexamined and reduced into a quotation, in order to summarise the content of each meaning unit.

Finally, the contents of the condensates were synthesised to summarise the findings that were related to the research question. Analytic texts of each code group were formulated to present the results and reflect the original context.

### Ethical considerations

Informed consent was obtained from all participants before the interviews. The participants received both written and verbal information about the study, its purpose, and examples of questions that would be asked. Further, information about the right to withdraw at any time was communicated. Participants were given time to consider their participation and were encouraged to ask any questions they had about the study. To ensure anonymity and confidentiality, all names of participants were replaced with numbers.

## Results

Participants experienced that the Co-Work-Care model had led to improved quality of care in several ways. The analysis of the interviews and focus group discussion are presented in three codes that emerged through the Malterud inductive analysis.^19^

### A structured work approach

Participants perceived that the care managers contributed to more structure when working with people with CMD. Shared responsibility in booking appointments and deciding about follow-ups made the care more predictable and systematic. GPs perceived that fewer patients were missed out on follow-ups and more patients were assessed at an early stage:

*'We have a structure to pick up people that are on sick leave and work with them earlier on. I think that it’s been very good. She* [the care manager] *screens everyone on sick leave* […] *the care manager helps to hold the system together. Helps to keep everyone in their place.'* (Individual interview 2, woman)

Participants perceived the common structured plan as a fundamental part of the Co-Work-Care model, where both employers and patients should be engaged in forming this plan. It was stressed that the person-centred meeting clarified potential difficulties that patients had at their workplace, as well as appropriate arrangements to facilitate RTW. In addition, the timeframe for when patients could return to their workplace became clearer for all parties through the person-centred dialogue meeting.

### Competency of the care manager and the rehabilitation coordinator

Participants emphasised the importance of having the right person with the right qualifications in the position as the rehabilitation coordinator. As important qualities, participants emphasised characteristics such as having a salutogenic approach, an ability to form positive relationships, and a reassuring way when working with patients.

Participants expressed that rehabilitation coordinators could sometimes shorten or even prevent a sickness absence through problem-solving, well considered rehabilitation plans, or suggestions for workplace adjustments. The rehabilitation coordinator reinforced team competency by being well-prepared and knowledgeable concerning regulations about employer obligations and health insurance. GPs felt that they themselves did not have enough knowledge within these areas of expertise and lacked sufficient time to learn and stay updated. Participants appreciated that the rehabilitation coordinator could fill these knowledge gaps:

*'The rehabilitation coordinator has a good relationship with the employers and is clear about their responsibilities. She is very competent and knowledgeable about regulations and each person’s role.*' (Focus group)

Some participants expressed that having the same person as both the rehabilitation coordinator and the care manager had several advantages. Their experience was that most communication around the patients was smoother and more effective:

*'The mental and psychosocial health is very important for the rehabilitation. Otherwise, there are two parallel tracks where you have to talk to the CM* [care manager] *and RC* [rehabilitation coordinator] *about days and dates. I don’t know. It’s like three parallel tracks. And then there is the physical therapist, CBT-therapist. There are too many contacts and parallel tracks.*' (Focus group)

Another participant perceived that the care manager had a clear role in the caretaking of patients with CMD. It was perceived as a great relief, knowing that the patients were in good hands.

### Gaining control through close collaboration

Participants described a feeling of increased job satisfaction since the Co-Work-Care model had been implemented. As a result, participants felt that they were able to dedicate more time to the patient’s medical conditions, which they saw as highly beneficial for the patients:

*'When you receive help from others, one gets a chance to focus on the doctor's role. It is very helpful when you meet the patient … Sometimes you feel that so much time needs to be spent on what to write in the certificate, that you don't have time for the medical consultation. So it’s really helpful to get this information from others.*' (Individual interview 2, woman)

Overall, participants perceived that the extensive collaboration had decreased the risk of missing important aspects of the care. Various issues were now evaluated simultaneously and in a structured manner, which was expected to also increase patient safety. Further, GPs explained that rehabilitation coordinators contributed to quality improvements by systematically screening patients on sick leave and assessing their rehabilitation needs. It was also emphasised that the rehabilitation coordinator was available during the entire sick leave process and could easily be contacted.

According to participants, collaboration with external parties such as the Swedish Social Insurance Agency had previously been burdensome. Participants expressed that sharing this sort of administrative work contributed to a less strained work situation. The rehabilitation coordinator also helped out with the assessments of disability and work ability of the patients, on which the GPs could base their medical certificates. All this help was perceived as relieving and time-saving:

*'I think that one is not as exhausted, one doesn’t feel like, "Oh no, now this falls on me." I take care of the medical things, and don’t have to hold everything together … And it’s very relieving to hand over those bits.'* (Focus group)

However, one of the GPs indicated that the collaboration would improve if doctors also participated during the meetings with the employer and expressed a willingness to engage more in each patient’s situation. According to participants, an important advantage with the rehabilitation coordinator was the strengthened collaboration with the patient’s employer. The rehabilitation coordinator made sure to engage the employer early in the rehabilitation process, which was considered important in order to elaborate a common plan:

*'I think that many employers have been really positive to be a part of this* [about collaboration]*. It becomes clearer to the patient that the workplace is an important part of the process …'* (Individual interview 3, man)

Participants stressed that the Co-Work-Care model contributed to an increased awareness among employers about their responsibilities during the rehabilitation process. Additionally, participants had observed a decline in sickness absence among their patients but were uncertain whether this was owing to the Co-Work-Care model or to other factors.

## Discussion

### Summary

This study explored GPs’ experiences of enhanced collaborative care organised at the PCC, which was part of the Co-Work-Care model, which included a person-centred dialogue meeting in the care of patients with CMD who needed sick leave certification. A main finding was that GPs felt that collaboration with a care manager and a rehabilitation coordinator had positively influenced their overall work situation in several different ways. Participants perceived that the enhanced collaboration reduced a large part of their workload and led to improved quality of their medical care regarding mental, as well as physical, health. Further, the GPs perceived that patients’ rehabilitation had become more effective since the introduction of the Co-Work-Care model.

Participants experienced that the introduction of the collaborative work, together with the care managers and rehabilitation coordinators, led to better patient safety and better care. This experience confirmed findings from previous research,^16^ showing that patients who had access to a care manager reported more positive experiences of the received care.

A recent article raised the issue that the support from rehabilitation coordinators varies between PCCs, and the authors concluded that this might mean that the quality of care becomes unequal.^20^ The rehabilitation coordinator working autonomously was perceived positively by most of the participants. However, some GPs indicated that collaboration would improve further if doctors were more engaged in the patient's situation; for example, by participating in the person-centred meeting with the employer.

In the present study, GPs experienced close collaboration with the rehabilitation coordinators and care managers. It has been argued that trust is one of the most central elements in a successful collaboration, although it takes time to develop.^21,22^ The GPs stressed repeatedly the importance of working with competent and collaborative care managers and rehabilitation coordinators. It seemed that collaborative work was person-dependent, which was also confirmed by physicians in an earlier study.^20^ Individual factors — for example, the willingness to collaborate and mutual trust among the professionals — were considered to be of even greater importance than systemic and organisational structures.^21–23^

However, to achieve a well-functioning collaboration, stability of the team over time is required. Unfortunately, many PCCs in Sweden are characterised by a high rate of turnover among healthcare personnel and thereby lack optimal conditions for developing a consistency of collaboration. For example, an earlier study showed that rehabilitation coordinators perceived the fact that GPs often switched workplaces as a main barrier to a more profound collaboration.^4^

### Strengths and limitations

The use of both individual interviews and a focus group enabled a broader and in-depth analysis. The study was carried out during the COVID-19 pandemic. The focus group discussion was therefore performed using a videoconference program. This is a potential limitation, since more active discussions might have been achieved if participants had met in person.

### Comparison with existing literature

Previous studies emphasised the importance of improving the rehabilitation process, partly owing to the fact that prolonged periods of sick leave decreased the chances of returning to the workplace.^2^ Participants in the current study perceived that a strengthened collaboration with the employer through the person-centred dialogue was one of the most important advantages of the Co-Work-Care model. This was also found in a previous interview study of the experiences of care managers and rehabilitation coordinators in the Co-Work-Care study.^5^ Moreover, participants argued that early engagement with employers had a clear positive influence on the RTW process, which was in line with the same study of the role of the rehabilitation coordinator.^4^

Participants appreciated when professionals of the group performed tasks commensurate with their own expertise. As shown earlier,^22,24^ mutually beneficial interdependency among the team members gives meaning to the different roles and increases willingness to participate in a collaboration. Previous research^24,25^ showed that a proper amount of autonomy among different professions in the team was considered necessary for a well-functioning collaboration. Conflicting interests — for example, among the social insurance office, the employers, and the healthcare systems — may negatively influence the RTW process.^2,26–29^

### Implications for research and practice

In general, participants in the current study experienced lack of time and a heavy workload as barriers to becoming more aware of the patient's situation. Similar results were shown in previous studies evaluating GPs’ experiences of how the role of the rehabilitation coordinator worked in actual work situations at the PCC.^10,30^ In addition, physicians in a recent focus group study^20^ requested more time for medical tasks and wanted less of their time to be spent on handling difficult sickness leave certifications and insurance policy questions.

GPs were relieved that rehabilitation coordinators could take over a part of collaboration with external parties, such as the Swedish Social Insurance Agency, which previously was experienced as burdensome and stressful. The results have indicated that GPs experienced a more sustainable work situation since the collaboration with rehabilitation coordinators and care managers was implemented.

## Data

The dataset relied on in this article is available from the corresponding author on reasonable request.
